# Unpacking Consumer Purchase Intentions Toward Plant-Based Meat Alternatives: An Integrated TPB–VAB Approach Using PLS-SEM, fsQCA, and NCA

**DOI:** 10.3390/foods14203525

**Published:** 2025-10-16

**Authors:** Jialiang Pan, Kun-Shan Wu, Hui-Ting Liu

**Affiliations:** 1 School of Business and Management, Jiaxing Nanhu University, Jiaxing 314001, China; 220055@jxnhu.edu.cn; 2 Department of Business Administration, Tamkang University, New Taipei City 251301, Taiwan; 411610214@o365.tku.edu.tw

**Keywords:** plant-based meat alternatives, purchase intention, PLS-SEM, fsQCA, necessary condition analysis

## Abstract

Plant-based meat alternatives (PBMAs) are gaining momentum in response to rising demand for sustainable and healthier diets. Drawing on an integrated framework combining the Theory of Planned Behavior (TPB) and the Value–Attitude–Behavior (VAB) model, this study explores key determinants shaping consumers’ purchase intention towards PBMAs in Taiwan. This study performed Partial Least Squares Structural Equation Modelling (PLS-SEM), fuzzy-set qualitative comparative analysis (fsQCA) and necessary condition analysis (NCA) to evaluate the formation of consumers’ PBMA purchase intention. The PLS-SEM results revealed that both environmental consciousness and health consciousness exert a significant influence on consumer attitudes, which, together with subjective norms and perceived behavioral control, positively predict purchase intention. fsQCA revealed six distinct combinations of conditions leading to high purchase intention, while NCA identified environmental consciousness, health consciousness, and the three TPB components as necessary conditions. The results highlight the mediating role of attitude and underscore the value of integrating multiple analytical perspectives to capture the complexity of consumer decision-making. This research advances both theoretical understanding and practical application by elucidating the psychological mechanisms underpinning PBMA adoption and by providing evidence-based implications for strategic marketing within the plant-based food sector.

## 1. Introduction

Dietary choices exert a profound impact not only on individual health but also on climate change and the wider sustainability of the planet [1]. Food production accounts for roughly one-third of global greenhouse gas (GHG) emissions, with meat production responsible for more than half of this figure [2]. Transitioning towards plant-based alternatives could reduce such emissions by almost half [3], prompting major food corporations such as Subway, Burger King, and Starbucks to expand their plant-based offerings as part of their sustainability strategies [4,5].

Plant-based diets, which consider any food category from animals off-limits, are deemed appropriate interventions in response to the issues of the environment, animal cruelty, and human health in a one-package deal [6]. Along with this, the sustainable consumption and production goals are integrated under the United Nations Sustainable Development Goals (SDGs), centered around SDG 2 (hunger eradication), SDG 3 (health and wellbeing), and SDG 12 (sustainable production and consumption) [7]. Thus, consumer focus has been directed towards plant-based products in line with these desires, and the global market is estimated to reach a value greater than USD 77 billion by 2025 [8]. Throughout all of this, it has been necessary to interrogate how consumers react to the implementation of plant-based meat alternatives (PBMAs), both from an academic and business standpoint [9,10].

According to the well-established studies, a range of variables influence plant-based eating spans from personal health beliefs to product characteristics, from social attitudes to moral or environmental issues [10,11,12,13,14]. Nevertheless, a significant area that remains unclear in these studies is the use of broad motivational factors, as they tend to divide and do not account for the larger picture of PBMA consumption. The Theory of Planned Behavior (TPB) has been, so far, a very suitable framework for examining consumer behavior in this area and for identifying these variables [15,16,17,18,19,20,21,22]. Nonetheless, there is still a gap remaining in the empirical findings. For instance, it is true that the validity of attitudes, norms, and perceived behavioral control has been reported in many studies [20,23,24]. However, this seems to be contradicted by the results published by other studies that emerged with different numbers, significantly, or opposite effects [24,25,26,27,28]. This once again brings the issue of the unexplained psychological complexities of PBMA buying behaviors to the fore.

Moreover, scholars increasingly argue that the TPB alone may be insufficient to account for the complex, value-driven nature of food-related decisions [18,19,29,30]. The Value–Attitude–Behavior (VAB) model complements TPB by incorporating underlying personal values, such as altruistic (environmental consciousness) and egoistic (health consciousness) orientations, which have been shown to shape pro-environmental and ethical consumption [31,32,33,34,35]. Integrating the TPB and VAB models can therefore provide a more comprehensive theoretical lens through which to examine consumers’ behavioral intentions towards PBMAs [36,37,38].

Methodologically, most existing research has employed symmetric analytical techniques such as partial least squares structural equation modelling (PLS-SEM), which assume linear and additive relationships among variables [5,7,14,20,22,23,25,26,27,39]. This linear perspective may fail to capture the complex, configurational nature of consumer decision processes [1,40]. To overcome this limitation, a hybrid methodological approach coupling PLS-SEM with fsQCA (fuzzy-set qualitative comparative analysis) and NCA (necessary condition analysis), is applied to allow for a more comprehensive and detailed study of both sufficient and necessary conditions underlying PBMA purchase intentions.

Collectively, this work aims to fill a theoretical and methodological void within the literature by striving: (1) To determine how environmental and health consciousness affect consumers’ attitudes towards PBMAs; (2) To explore how attitude, subjective norm, and perceived behavioral control influence purchase intention; (3) To examine the mediating role of attitude in connecting environmental and health consciousness variables with behavior intention; and (4) To find both sufficient conditions and necessary conditions of factors for driving PBMA purchase intention through an integrative framework of PLS–fsQCA–NCA.

This study offers a holistic understanding of the process of sustainable dietary transition and adds value to sustainable consumption theory by combining the value-based and behavioral perspectives, as well as practical implications for policymakers and industry in the shift toward more sustainable dietary transitions.

## 2. Conceptual Background

### 2.1. Extended the Theory of Planned Behavior

According to the Theory of Planned Behavior (TPB), human actions are chiefly governed by attitudes (ATT), subjective norms (SN), and perceived behavioral control (PBC). This framework offers a robust theoretical foundation for elucidating the cognitive mechanisms that underpin individual intentions and consumer purchasing decisions [15]. This model has been extensively applied in research on PBMA consumption behavior [10,22,24,25,26,27,41,42].

Complementing TPB, the Value–Attitude–Behavior (VAB) model, proposed by Homer and Kahle [43], asserts that consumer values significantly shape attitudes, which subsequently influence behavior [44]. The VAB framework has shown considerable explanatory power in predicting environmentally responsible food consumption and purchasing behavior towards PBMAs [45]. Drawing on recent studies in the domain of plant-based food consumption [5,23], this paper conceptualizes health consciousness as an egoistic value—centered on personal benefit—and environmental consciousness as an altruistic value—motivated by concern for the broader ecological good. By incorporating these constructs within the TPB framework, this article seeks to enhance our understanding of the cognitive and motivational determinants that underpin consumers’ purchase intentions towards PBMAs. This integrative perspective facilitates a more nuanced examination of the complex interplay between values, attitudes, and behavioral intentions, as outlined in the subsequent sections.

### 2.2. Environmental Consciousness

Environmental consciousness is inherently altruistic, as it reflects behavior motivated by the desire to protect the natural environment without expectation of personal gain [46]. Such consciousness is closely associated with consumers’ altruistic values and pro-environmental purchasing considerations, whereby individuals choose PBMAs primarily for their perceived environmental benefits. Environmental consciousness refers to the extent to which individuals are aware of environmental issues and actively engage in actions to mitigate them [47], and has emerged as a pivotal determinant of PBMA consumption preferences [23]. Within the context of sustainable meat alternatives, prior research has demonstrated that environmental consciousness serves as a particularly strong predictor of consumers’ attitudes [23]. Empirical evidence supports its positive influence on consumers’ evaluative orientations towards these products [48]. Likewise, recent studies [5,49] further corroborate that heightened environmental consciousness significantly fosters favorable attitudes towards PBMAs.

Moreover, environmental consciousness has been widely recognized as a salient determinant influencing consumers’ attitudes, intentions, and behaviors towards organic food purchases [50,51]. Findings reported by Miftari et al. [52] further corroborate the significant and positive influence of environmental consciousness on consumers’ purchase intentions towards organic food. More recently, Li and Shan [53] also found that environmental consciousness exerts a positive effect on consumers’ intentions to purchase green-packaged organic products. On the basis of the aforementioned evidence, the following hypothesis is formulated:

**H1.**  *Environmental consciousness exerts a positive influence on consumers’ attitudes towards PBMAs.*

**H2.**  *Environmental consciousness exerts a positive influence on consumers’ purchase intention towards PBMAs.*

### 2.3. Health Consciousness

Health consciousness reflects a pro-self orientation, encompassing individuals’ concern for their own well-being and that of their families, and is therefore regarded as egoistic in nature [54]. Compared with conventional food products, PBMAs are commonly perceived as a healthier option, largely due to the absence of harmful fertilizers in their production processes. Health consciousness refers to an individual’s assessment of their willingness and readiness to engage in health-promoting behaviors [55]. Consumers commonly associate plant-based foods with health benefits [56]; therefore, individuals with higher health consciousness are more inclined to reduce animal-based food consumption in favor of PBMAs [57].

The role of health consciousness in shaping consumer attitudes has been extensively explored in the food consumption literature, especially concerning decisions related to nutrients, healthy and sustainable eating behaviors. For instance, Kumar [58] demonstrated that consumers’ health consciousness significantly shapes their cognitive attitudes towards health-oriented food products. Similarly, Munaqib et al. [59] identified health consciousness as a significant predictor of consumers’ intentions to engage in environmentally responsible purchasing behavior. Within the context of PBMAs, an expanding body of research has similarly established a robust positive association between health consciousness and favorable consumer attitudes towards PBMAs [5,60,61].

Moreover, increasing public awareness of health concerns has fostered a cohort of highly health-conscious consumers who assess products both for their direct health benefits and the environmental sustainability of their ingredients and production processes [62]. Consequently, such consumers display a marked preference for products that align with both health and ecological values. Many studies have shown that health consciousness is a major factor that directly increases consumers’ intention to buy green products, especially in health-related areas such as organic foods and wellness products [63,64,65,66]. Furthermore, heightened health consciousness not only fosters more favorable attitudes towards environmentally responsible products but also facilitates the translation of these attitudes into tangible purchasing behavior [67]. In light of the aforementioned evidence, the following hypothesis is formulated:

**H3.**  *Health consciousness exerts a positive influence on consumers’ attitudes towards PBMAs.*

**H4.**  *Health consciousness exerts a positive influence on consumers’ purchase intention towards PBMAs.*

### 2.4. PBMA Purchase Intentions Driven by Three TPB Constructs

According to the TPB model, behavioral intention can be predicted by an individual’s attitude (ATT) towards the behavior, subjective norms (SN), and perceived behavioral control (PBC) [15]. TPB has consistently demonstrated its robustness as a theoretical framework for predicting consumer behavior in the context of PBMA consumption [10,24,26,27,42]. Seo et al. [24] confirmed that all three TPB components (ATT, SN, and PBC) positively influence Korean consumers’ PBMA purchase intention. Similarly, Li et al. [20] reported that these three TPB constructs significantly and positively influence the purchase intentions of Taiwanese consumers towards plant-based egg products. Nguyen et al. [68] documented that three TPB constructs significantly and positively influence customers’ intention to purchase sustainable products. Drawing on these theoretical insights, the following hypotheses are proposed:

**H5.**  *Attitude positively affects consumers’ purchase intention towards PBMAs.*

**H6.**  *Subjective norms positively affect consumers’ purchase intention towards PBMAs.*

**H7.**  *Perceived behavioral control positively affects consumers’ purchase intention towards PBMAs.*

### 2.5. Mediating Role of Attitude

Empirical evidence consistently demonstrates that attitudes towards organic foods play a mediating role in the relationship between purchase behavior and both environmental and health consciousness [69,70,71]. Recent findings by Pan and Wu [65] found that purchase intentions for organic foods are significantly shaped by health consciousness through the mediating role of attitude. Consistent with this, Pan et al. [66] further indicated that health consciousness exerts a strong positive influence on consumers’ purchase intentions towards health and wellness foods, with attitude serving as a key mediating variable. Likewise, according to Li and An [72], both health and environmental consciousness directly shape consumers’ intentions to purchase sustainable sports products, while simultaneously exerting an indirect effect mediated by attitude. Correspondingly, Li and Shan [53] demonstrated that health and environmental consciousness significantly shape consumers’ purchase intentions for green-packaged organic foods, operating through both direct effects and mediated pathways. In light of the aforementioned empirical evidence, the following hypotheses are proposed:

**H8.**  *Consumers’ attitudes towards PBMAs mediate the relationship between environmental consciousness and purchase intention.*

**H9.**  *Consumers’ attitudes towards PBMAs mediate the relationship between health consciousness and purchase intention.*

**Figure 1:**
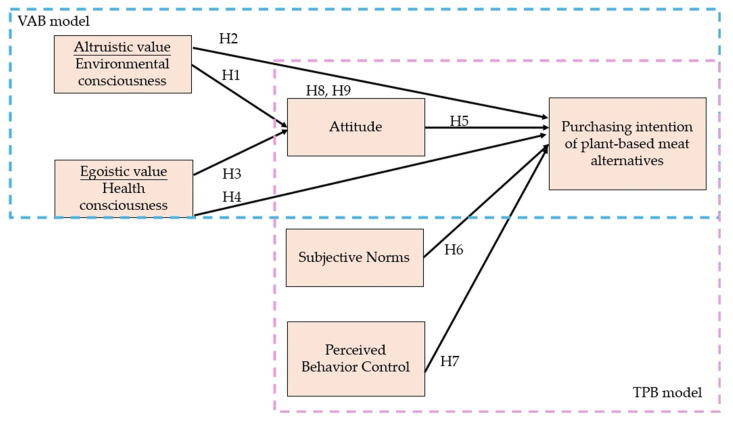
Conceptual framework and study hypotheses.

Figure 1 presents the proposed conceptual model that encapsulates the theoretical framework of this study.

## 3. Materials and Methods

### 3.1. Research Instruments

In this study, the survey instrument was developed by adapting and extending established measurement items to ensure content validity. Specifically, environmental consciousness (four items), health consciousness (four items), attitude (three items), and purchase intention towards PBMAs (three items) were drawn from Park and Namkung [5], while subjective norms (three items) and perceived behavioral control (three items) were adapted from Chen et al. [26]. The complete list of items and their original sources is provided in Appendix A. All items were measured using a five-point Likert scale (1 = strongly disagree; 5 = strongly agree). To ensure content validity throughout the translation process from English to Chinese, the back-translation technique was employed. Additionally, three experts—including practitioners in the plant-based meat alternative industry and university academics—reviewed the items to confirm that they accurately captured the intended constructs. Moreover, a pilot test with 30 prospective participants was conducted to evaluate the reliability of the measurement constructs. The finalized questionnaire is provided in Appendix A.

### 3.2. Participants and Procedure

Conducted in July 2025, this cross-sectional study tested the proposed hypotheses using data collected through an online survey. The questionnaire was distributed via SurveyCake (https://www.surveycake.com/), a widely used online survey platform in Taiwan, and disseminated through the Line messaging platform to reach participants efficiently. Convenience sampling was adopted, as it provides a rapid, cost-effective, and practical means of recruiting readily accessible respondents [73]. Participants who self-identified as meat-eaters, specifically individuals who regularly consume animal-based red meat, were invited to participate in this study.

In alignment with ethical research standards, participation in this study was entirely voluntary. Participants were provided with comprehensive information outlining the purpose and scope of the research, alongside clear assurances of their right to withdraw at any stage without consequence. All personal and professional identifiers were removed to preserve anonymity and confidentiality. Furthermore, the questionnaire was carefully designed to minimize the potential for psychological discomfort or distress, thereby fostering a respectful and ethically sound research environment.

**Figure 2:**
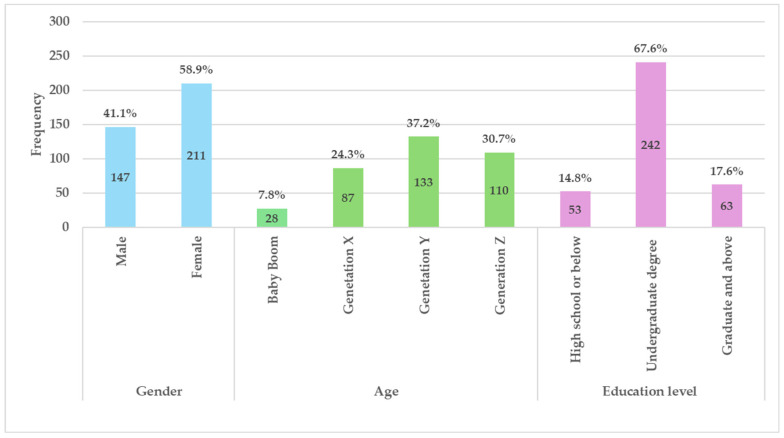
Sample demographic characteristics (*n* = 358).

Altogether 400 questionnaires were collected, 42 questionnaires were excluded due to either incomplete responses or being completed in an implausibly short duration (i.e., under 100 s). This resulted in 358 valid questionnaires for data analysis, yielding a valid response rate of 89.5%. Figure 2 displays the demographic profile of the respondents. Of the sample, 58.9% were female, while 37.2% belonged to Generation Y. Furthermore, approximately 67.2% of participants had attained an undergraduate degree.

### 3.3. Statistical Analysis

This study employed both symmetric and asymmetric analytical approaches to identify the causal configurations of antecedents and the key conditions leading to the desired outcome. For the symmetric analysis, we adopted the PLS-SEM (partial least squares structural equation modeling) technique. Relying solely on symmetric methods like PLS-SEM may fail to capture the complexity of asymmetric relationships, particularly in applied sustainable consumption research contexts [1]. To address this limitation, researchers are encouraged to complement symmetric approaches with fsQCA (fuzzy set qualitative comparative analysis) [74]. fsQCA can accommodate non-linear associations [75], highlighting that a unit change in a predictor does not necessarily lead to a constant change in the outcome. While fsQCA excels at identifying diverse causal configurations and detecting whether a condition is necessary in kind for an outcome, its scope is limited to necessity in kind alone [76]. In contrast, NCA (necessary condition analysis) extends this capability by examining both the kind and degree of necessity, thus offering a richer analytical perspective [77]. Specifically, NCA identifies necessary conditions, estimates their effect sizes, and pinpoints bottleneck thresholds that must be met to achieve varying levels of the outcome variable [78]. The following sections present and discuss the findings obtained from the PLS-SEM, fsQCA, and NCA analyses.

## 4. Analysis and Results

### 4.1. Common Method Bias

This article employed three approaches to assess potential common method bias (CMB). First, procedural remedies were implemented in line with the guidelines proposed by Podsakoff et al. [79], aiming to reduce self-reporting bias. These included assuring respondents of anonymity and confidentiality, as well as randomizing the order of questionnaire items to mitigate potential order effects.

Second, Harman’s single-factor test revealed that the first unrotated factor accounted for 47.709% of the total variance—close to, yet below, the conventional 50% threshold. However, as this criterion is not definitive and the test itself has limited diagnostic power [80,81], two additional statistical techniques were employed: the marker variable approach [82] and the unmeasured latent method construct (ULMC) test [83].

Substantive factor loadings were all statistically significant, whereas the method factor loadings were non-significant. The ratio of substantive to method variance (250:1) indicated that the CMB was negligible (Table 1). Furthermore, following Lindell and Whitney’s [82] guidelines, a theoretically unrelated marker variable [84] was introduced; the inclusion of this variable did not result in a meaningful increase in *R*^2^. Finally, the highest observed correlation between constructs (ATT and PI = 0.745) was below the accepted collinearity threshold of 0.8 [85,86]. Collectively, these results confirm that CMB did not materially affect the study’s findings.

### 4.2. PLS-SEM Results

#### 4.2.1. Assessment of Measurement Model

The data were analyzed using PLS-SEM via SmartPLS 4. As exposed in Table 2, the factor loadings for all constructs—environmental consciousness (EC), health consciousness (HC), attitude (AT), subjective norms (SN), perceived behavioral control (PBC), and purchase intention (PI)—exceeded the recommended threshold of 0.70, as advised by Hair et al. [87]. Also, the composite reliability (CR) values ranged from 0.889 to 0.930, substantially surpassing the commonly accepted benchmark of 0.70, thereby demonstrating robust internal consistency across the measurement model. Furthermore, the average variance extracted (AVE) values lie between 0.667 and 0.815, comfortably exceeding the minimum recommended level of 0.5, thereby confirming the constructs’ convergent validity.

Additionally, the heterotrait–monotrait (HTMT) ratio was employed to assess discriminant validity, in preference to traditional approaches such as the Fornell–Larcker criterion [88]. The analysis revealed that all HTMT values fell below the conservative threshold of 0.9 [87], thus providing strong evidence of discriminant validity (Table 3).

#### 4.2.2. Structural Model Assessment

**Figure 3:**
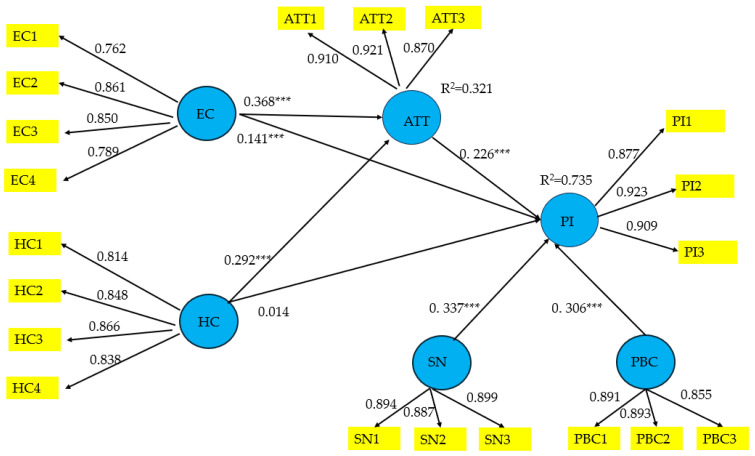
Overview of the model estimates. Note: *** *p* < 0.01. ATT = Attitude, EC = Environmental consciousness, HC = Health consciousness, PBC = Perceived behavior control, SN = Subject norms, PI = Purchase intention.

The structural model hypotheses were evaluated using the bias-corrected and accelerated (BCa) bootstrap procedure, based on 10,000 resamples. The results (Figure 3 and Table 4) reveal that EC exerts a significant positive influence on both ATT and PI. HC also demonstrates a positive effect on ATT; however, its direct influence on PI is not statistically significant. Moreover, ATT is found to have a strong positive association with PI. In addition, both SN and PBC are shown to exert significant positive effects on consumers’ PI towards PBMAs.

The model’s *R*^2^ values, representing the coefficient of determination, specifically the model accounts for variances of 73.5% in purchase intention towards PBMAs, and 32.1% in attitude, indicating robust explanatory power [89]. The effect sizes (*f*^2^) fell within the interval of 0.049–0.091. Similarly, the model’s predictive relevance, evaluated via *Q*^2^ values, ranged from 0.310 to 0.692, thereby demonstrating robust predictive capability across the dataset [89]. Moreover, the model’s overall fit was evaluated using the Standardized Root Mean Square Residual (SRMR), which is widely recognized as a robust and reliable indicator of model adequacy. The SRMR serves as a diagnostic tool to detect potential model misspecifications within PLS-SEM frameworks [90]. Conventionally, an SRMR value of 0.08 or lower is regarded as indicative of a satisfactory model fit [91]. In the present study, the SRMR was calculated at 0.054, falling well below the recommended threshold, thereby confirming that the structural model demonstrates an acceptable and adequate fit to the data. These results collectively underscore the robust predictive power of the proposed research framework.

Additionally, mediation analyses were performed to examine the indirect effects among variables within the proposed theoretical framework. The results (Table 4) reveal that attitude partially mediates the relationship between environmental consciousness and purchase intention, while it fully mediates the relationship between health consciousness and purchase intention. Thus, attitude plays a pivotal role in shaping purchase intention.

### 4.3. fsQCA Results

The fsQCA procedure comprises three distinct phases: data calibration, analysis of necessary conditions, and assessment of sufficient conditions.

#### 4.3.1. Data Calibration

Data calibration is a crucial step in fsQCA, as it transforms raw numerical data into set membership scores using qualitative breakpoints. As the data in this study were derived from questionnaires comprising multiple indicators per construct, the indicator scores for each construct were initially aggregated, followed by the computation of their respective mean values [40].

During the calibration phase, three qualitative anchor points were employed to perform a fine-grained calibration of membership scores for each case within the relevant sets, thereby enabling the transformation of raw data into fuzzy-set membership values in accordance with established procedures [40]. Given that our study used a 5-point Likert scale, various methods for calibrating such data have been proposed in previous literature. Following the approach recommended by Pappas and Woodside [40], we established three thresholds: 0.05 (full non-membership), 0.5 (crossover point), and 0.95 (full membership) to determine fuzzy set membership (see Table 5). Using these established thresholds, the data were calibrated within the fsQCA 4.1 software to generate fuzzy sets with membership scores ranging from 0 to 1. In the final stage of the calibration process, any case with a membership score of exactly 0.5 was adjusted to 0.501, in accordance with the recommendation by Fiss [92]. This modification was implemented to circumvent potential analytical complications inherent in fuzzy set operations, as scores of precisely 0.5 may hinder the accurate computation of set-theoretic intersections [93].

#### 4.3.2. Analysis of Sufficient Conditions

Following Ragin’s [93] approach, a truth table was generated to identify condition configurations sufficient for the outcome of interest (refer to Table 6). The fsQCA procedure yields three solution types: complex, parsimonious, and intermediate. This study prioritized the intermediate solutions, as they offer a balanced approach between empirical complexity and theoretical parsimony, thereby providing a more accurate representation of the observed cases. Additionally, we supplemented this analysis by comparing the intermediate solutions with the simple (parsimonious) solutions and conducting peripheral analyses to deepen our interpretation of the findings. Through these steps, we derived the sufficient configurations of antecedent conditions that lead to the desired outcome.

Table 6 presents detailed results, revealing six distinct configurations that lead to high purchase intention towards PBMAs. The high overall coverage value of 0.939 indicates that these six configurations explain a substantial proportion of purchase intention towards PBMAs. Furthermore, the overall solution consistency score of 0.772—exceeding the commonly accepted threshold of 0.75 [40]—attests to the reliability and robustness of the identified configurations in predicting purchase intentions towards PBMAs.

Based on Table 6 (6A), several alternative configurations can lead to high purchase intention towards PBMAs. Solution 1 shows that even with low levels of health consciousness, a high level of attitude is sufficient to generate strong purchase intention. Solution 2 indicates that high attitude combined with high perceived behavioral control also predicts greater purchase intention. Solution 3 demonstrates that simultaneously high levels of attitude and subjective norms can drive higher purchase intention. Solution 4 reveals that high subjective norms, together with low environmental and health consciousness, can likewise lead to high purchase intention. In comparison, solution 5 suggests that higher environmental and health consciousness paired with lower perceived behavioral control can still produce elevated purchase intention. Finally, solution 6 highlights that higher environmental consciousness and perceived behavioral control, despite low subjective norms, can also result in greater purchase intention towards PBMAs.

On the flip side, six causal configurations (coverage: 0.883, consistency: 0.818) were identified as leading to low purchase intention towards PBMAs (Table 6 (~6A)). Specifically: Solution 1: low environmental consciousness and low health consciousness (~EC *~HC); Solution 2: high environmental consciousness combined with low subjective norms (EC *~SN); Solution 3: low attitude, low subjective norms, and low perceived behavioral control (~ATT *~PBC *~SN); Solution 4: high environmental consciousness and health consciousness coupled with low perceived behavioral control (EC *HC~* PBC); Solution 5: low environmental consciousness together with high attitude and high perceived behavioral control (ATT~EC * PBC); and Solution 6: low perceived behavioral control along with high attitude and high subjective norms (ATT~PBC * SN). Collectively, these configurations illustrate the complex and asymmetric pathways that can lead to diminished purchase intention towards PBMAs.

### 4.4. Necessary Condition Analysis Results

**Figure 4:**
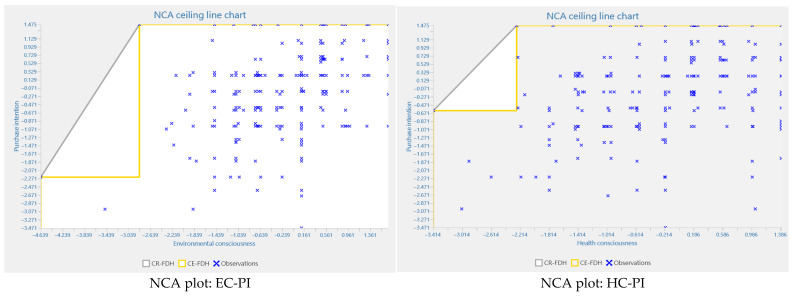

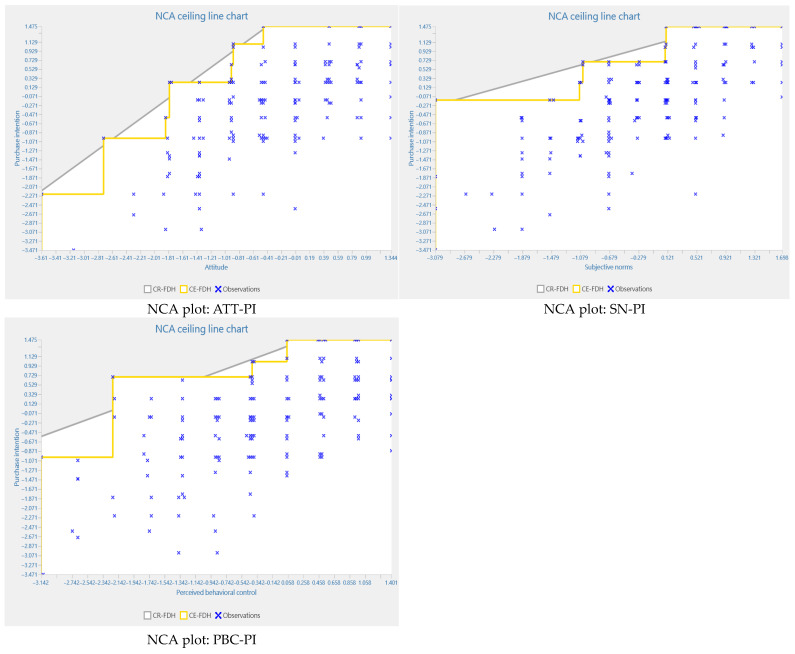
Scatter plots of the predictor variables versus purchase intention towards PBMAs.

We employed necessary condition analysis (NCA) [94] to conduct a robust test of necessity. In accordance with the suggestions of scholars [76,95] and in light of the presence of irregular data patterns near the ceiling boundaries, this study adopted the ceiling envelopment–free disposal hull (CE-FDH) method, owing to its demonstrated precision and suitability under such conditions. The analysis used the CE-FDH approach with 10,000 bootstrap samples per condition to determine the *p*-values. Figure 4 depicts the scatter plots between each predictor and purchase intention towards PBMAs.

According to Dul et al. [77], a variable must satisfy three criteria to be considered a necessary condition: (1) theoretical justification for its necessity, (2) an effect size exceeding 0.1, and (3) a statistically significant *p*-value (*p* < 0.05). As presented in Table 7, all latent constructs—environmental consciousness, health consciousness, attitude, subject norms, and perceived behavioral control—fulfilled these requirements, with effect sizes exceeding the 0.1 threshold and *p*-values below 0.05. All were identified as significant necessary conditions with medium effect sizes.

To provide deeper insights, we conducted a bottleneck analysis (Table 8). This analysis identifies the minimum threshold levels required for each independent variable (e.g., attitude, subjective norms) to reach specified target levels of the dependent variable (purchase intention). The results show that achieving an 80% level of purchase intention requires an attitude to be at least 19.274% and subjective norms to reach or exceed 11.732%. In contrast, attaining the maximum purchase intention level (100%) demands higher prerequisites: a minimum attitude level of 27.654% and subjective norms of at least 53.352%. In other words, if these minimum thresholds—27.654% for attitude and 53.352% for subjective norms—are not met, achieving the highest level of purchase intention becomes unattainable.

### 4.5. Testing for Predictive Validity

**Figure 5:**
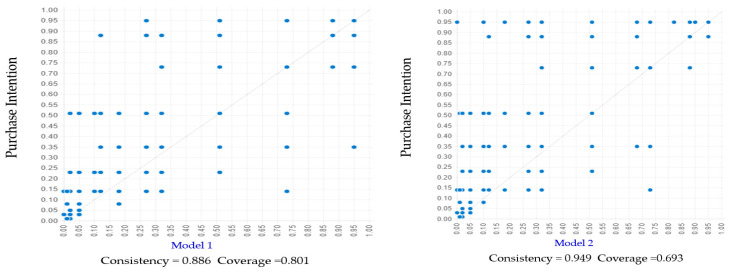

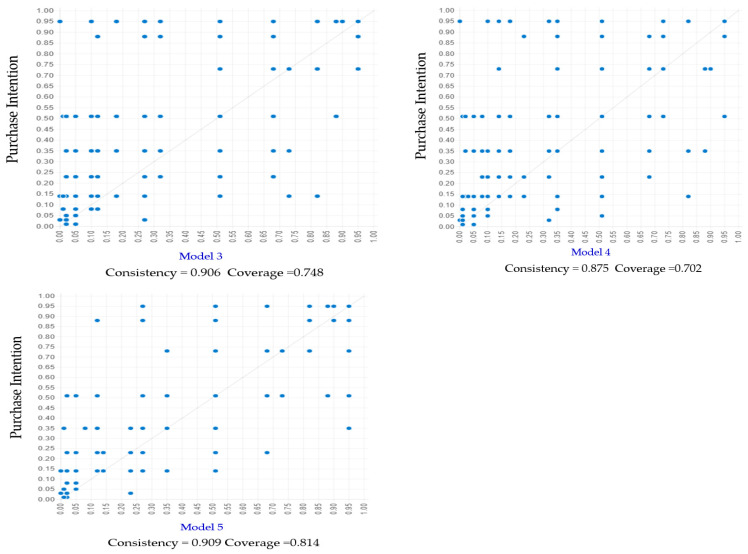
Fuzzy set plots for Models 1–5 based on subsample solutions, validated with holdout sample data.

In line with the latest guidelines proposed by Pappas and Woodside [40], this study assessed predictive validity by examining five configurational models derived from the subsample, as detailed in Table 9. The corresponding fuzzy plots, based on the holdout sample data, are presented in Figure 5. For instance, in Model 1 (Figure 5), the consistency score of 0.886 indicates a high level of consistency, and the coverage score of 0.801 suggests substantial explanatory power. These figures imply that Model 1 captures approximately 89% of consistent cases and represents 80% of the total membership in PBMA purchase intention, effectively making it a substantial subset of the outcome.

As shown in Table 9, the complex antecedent configurations exhibit strong predictive capacity for high purchase intention towards PBMAs. In line with Woodside [96], models with consistency values exceeding 0.80 are considered reliable and contribute meaningfully to theoretical advancement. Notably, when assessing predictive validity, it is expected that only the overall solution consistency and coverage for the subsample will be comparable to those of the full sample; the specific configurations themselves may differ [40].

The assessment of predictive validity across all five configurational models revealed both a high level of internal consistency within the subsample and considerable predictive accuracy when applied to the holdout sample, with reciprocal validation observed. Moreover, the overall solution consistency and coverage exhibited close alignment with those derived from the full dataset, thereby offering further empirical support for the robustness and generalizability of the identified models, as detailed in Table 9.

## 5. Discussion

Steered by the VAB and TPB models, this study examined how configurations of altruistic values (i.e., environmental consciousness), egoistic values (i.e., health consciousness), and the three central TPB constructs—attitude (ATT), subjective norms (SN), and perceived behavioral control (PBC)—collectively shape meat-eaters’ intentions to purchase PBMAs.

### 5.1. Findings of PLS-SEM

The principal finding of this paper is the positive influence of values—specifically health consciousness and environmental consciousness—on consumers’ attitudes towards PBMAs, which aligns with prior research [5,23,59]. The PLS-SEM analysis further validated that ATT, SN, and PBC each exert a significant positive effect on consumers’ PBMA purchase intention. These findings are consistent with earlier studies [10,20,26,27,68], thereby reinforcing the applicability of the TPB framework in this context.

Notably, this study highlights that subjective norms emerge as the strongest direct predictor of purchase intention towards PBMAs. This suggests that Taiwanese consumers tend to place considerable weight on the opinions and recommendations of others when deciding whether to purchase PBMAs. This finding underscores the significant role of social pressure and perceived expectations in sustainable consumption decisions [20]. Such a pattern may be deeply influenced by Taiwan’s multicultural context and sociocultural emphasis on interpersonal relationships and collectivist values [20].

This study also identified perceived behavioral control as a significant predictor of PBMA purchase intention. One possible explanation is that the sample primarily comprised individuals with higher education levels, who may possess greater environmental and health consciousness and feel more empowered to make autonomous decisions, such as choosing PBMAs. This result aligns with findings by Li et al. [20], which showed that individuals with higher educational attainment demonstrate a greater propensity toward adopting plant-based alternatives like plant-based eggs.

Furthermore, attitude is positively associated with purchase intention. Mediation analyses indicate that attitude partially mediates the relationship between environmental consciousness and purchase intention, while it fully mediates the relationship between health consciousness and purchase intention. These findings are consistent with earlier studies [10,20,26,27,68], thereby highlighting the central role of attitudes as a bridge linking personal values to actual behavioral intentions in the context of PBMAs choices.

### 5.2. Findings of fsQCA

PLS-SEM identified a single optimal model explaining consumers’ purchase intention toward PBMAs. In contrast, fsQCA challenged the PLS-SEM assumption that a single model can capture the behavior of all consumers by identifying six distinct configurations of factors leading to high purchase intention towards PBMAs. These six configurations collectively demonstrated an explanatory power of 93.9%, which is notably higher than the 72.1% explanatory power of the single PLS-SEM model. This finding underscores that a single symmetric model cannot fully account for the diversity of consumer behaviors. Given individual heterogeneity, consumers hold different motivations for purchasing PBMAs, and the fsQCA results more accurately reflect this complexity observed in the real world.

According to the fsQCA results, consumers with low environmental consciousness and health consciousness can still exhibit high PBMA purchase intention when influenced by strong subjective norms (Solution 4). This suggests that emphasizing and enhancing subjective norms should be prioritized to attract consumers who may otherwise lack intrinsic environmental or health motivations. Furthermore, the analysis indicates that consumers with low perceived behavioral control may nonetheless develop high purchase intention towards PBMAs when their environmental consciousness and health consciousness are strongly reinforced (Solution 5). These findings highlight the importance of tailored strategies that leverage different combinations of motivational factors to effectively promote PBMA adoption across diverse consumer segments.

### 5.3. Findings of NCA

The findings of NCA indicate that environmental consciousness, health consciousness, ATT, SN, and PBC each constitute necessary conditions for the formation of purchase intentions towards PBMAs. Bottleneck analysis further revealed that the relative importance of these factors varies across different levels of purchase intention. Specifically, attitude becomes the most critical factor when purchase intention reaches 80% or higher, whereas its importance diminishes at lower levels (≤70%). At these lower levels, subjective norms become the most influential driver.

These findings challenge the linear perspective offered by PLS-SEM, highlighting that the effects of these variables are non-linear and depend on the extent of purchase intention. Finally, to achieve the highest level of purchase intention (100%), subjective norms are the most decisive factor, followed by perceived behavioral control and attitude.

### 5.4. Compare and Contrast the Results Obtained from PLS-SEM, fsQCA, and NCA

A comparative analysis of results from PLS-SEM, fsQCA and NCA provides a more comprehensive understanding of consumers’ purchase intention towards PBMAs (see Table 10). The PLS-SEM analysis identified EC, HC, SN, PBC and ATT as significant predictors of purchase intention. ATT served as a partial mediator between EC and purchase intention and as a full mediator between HC and purchase intention. In contrast, the fsQCA results reveal multiple configurational pathways leading to high purchase intention, demonstrating that no single factor is sufficient in isolation. Rather, distinct combinations—such as high EC and ATT, or strong SN and PBC—can each generate high purchase intention, underscoring the causal asymmetry and complexity of consumer behavior. Complementing these findings, the NCA results show that EC, HC, ATT, SN and PBC are necessary conditions for high purchase intention, each exerting a medium-level effect. Collectively, these results highlight the complementary nature of the three analytical approaches: PLS-SEM elucidates the direct effects and mediation mechanisms, fsQCA uncovers alternative causal configurations, and NCA identifies the minimum conditions essential for high purchase intention.

## 6. Implications and Recommendations

### 6.1. Theoretical Implications

The findings of this study yield several significant theoretical implications. Firstly, by integrating the TPB with VAB model, this research advances a more holistic framework for elucidating the intricate interrelationships among key psychological constructs underpinning consumers’ purchase intentions towards PBMAs. Specifically, the study underscores the critical influence of both altruistic values (i.e., environmental consciousness) and egoistic values (i.e., health consciousness) in shaping consumer attitudes, which subsequently influence purchase intentions. This extended theoretical model contributes to the literature by offering a more holistic and nuanced understanding of individual behavior within the domain of sustainable consumption, particularly in the context of PBMAS.

Secondly, the concurrent application of PLS-SEM, fsQCA, and NCA in investigating the factors influencing consumer purchase intentions towards PBMAs enables the identification of both linear and non-linear causal relationships among variables. This multi-method strategy not only strengthens the robustness of the findings but also uncovers novel causal pathways through diverse configurational conditions. As a result, this integrative analytical framework offers a more holistic and nuanced understanding of consumers’ purchase intentions towards PBMAs, thereby contributing meaningfully to the existing literature on consumer behaviour within this context.

### 6.2. Managerial Implications

The findings of this study yield several managerial implications. Firstly, it is recommended that marketers strategically segment their target audiences according to distinct value orientations and attitudinal dispositions towards PBMAs. Consumers who exhibit high levels of environmental and health consciousness, along with a favorable attitude towards PBMAs, constitute a potentially loyal and engaged market segment. Developing tailored marketing strategies that align with the values and preferences of this group can enhance PBMAs’ message relevance and resonance. This targeted approach is likely to foster more consumer engagement and increase the effectiveness of promotional efforts.

Secondly, the environmental and health benefits of PBMAs have become key drivers of consumer purchasing decisions, contributing significantly to the growing adoption of plant-based diets. To capitalize on this trend, industry stakeholders—such as food service providers and product manufacturers—should consider offering behavioral nudges and transparent product information. For example, food service companies could provide detailed descriptions of PBMA menu items and emphasize the environmental advantages of PBMA production, such as reduced greenhouse gas (GHG) emissions, lower water consumption, and decreased land use. Furthermore, the use of labelling that emphasizes sustainability credentials may serve to strengthen consumer trust and exert a positive influence on purchasing behavior.

Thirdly, although demand for PBMAs continues to grow, a substantial proportion of consumers remain unaware of their actual health and environmental benefits. To bridge this informational gap, comprehensive educational and promotional initiatives should be undertaken to raise public awareness and deepen consumer understanding. Such efforts ought to clearly distinguish the production processes of PBMAs from those of conventional meat, while underscoring the associated positive health outcomes and environmental advantages.

Finally, this study identifies multiple, qualitatively distinct configurations of antecedent conditions that are associated with both elevated and diminished levels of purchase intention towards PBMAs, thereby highlighting the complexity and heterogeneity of consumer decision-making in this context. These insights hold significant implications for marketing strategy, as they underscore the importance for marketers and retailers to holistically consider consumer value before formulating targeted approaches. It is recommended that marketers take into account the complex and non-linear configurational effects identified in this research and develop flexible, evidence-informed strategies that align with the diverse and nuanced drivers of consumer purchase intentions towards PBMAs. As psychographic variables, consumer values provide a robust basis for identifying environmentally conscious market segments, thereby enabling more targeted and effective segmentation and positioning strategies.

### 6.3. Limitations and Future Works

Notwithstanding the valuable contributions of this study, its conclusions should be interpreted with caution owing to several limitations that warrant acknowledgement and further investigation.

Firstly, this study was confined to the Taiwanese market, which may restrict the generalizability of its findings to other cultural and geographical contexts. To enhance external validity and broaden the applicability of the results, future research is encouraged to adopt a more heterogeneous sampling strategy encompassing consumers from a range of regions or countries. Such an approach would facilitate cross-cultural comparisons and yield a more holistic understanding of consumer behavior towards PBMAs across diverse socio-cultural contexts.

Secondly, the use of a cross-sectional design constrains the capacity to infer causal relationships in the development of PBMA purchasing behavior. To address this limitation, future research is advised to adopt a longitudinal design in order to capture temporal changes in consumer values, attitudes, and behaviors. Such an approach would offer deeper insights into the causal mechanisms underlying PBMA adoption and allow for the observation of dynamic behavioral shifts in response to evolving social, environmental, or market factors.

Thirdly, the use of a convenience sampling method may limit the representativeness of the study’s findings. To enhance generalizability, future works should adopt more rigorous sampling methods, such as stratified sampling, to ensure that key subgroups are adequately represented based on relevant demographic or behavioral characteristics.

Fourthly, only data from meat-eaters were collected from this study, so the generalizability of the findings is limited as it only exhibits the viewpoints and purchase intentions of meat-eaters. The results cannot be generalized for non-meat consumers from different cultures; thus, future studies should investigate more diverse cultures. It is suggested that future studies survey different cultures’ consumers and perform comparison analyses or multigroup analyses.

Finally, although the study relied on self-reported behavioral intentions—which may not fully reflect actual purchasing behavior—it nevertheless offers valuable and highly relevant insights. Future research should therefore examine real consumption patterns to address the gap between stated intentions and observable consumer actions.

## 7. Conclusions

This study seeks to investigate the intricate pathways underpinning consumers’ PBMA purchase intentions in Taiwan by using a hybrid methodological approach of PLS-SEM, fsQCA, and NCA. In doing so, it addresses critical gaps in the current understanding of consumer decision-making related to plant-based dietary choices. From a theoretical perspective, the integration of the TPB and VAB model advances existing knowledge by capturing complex, configurational patterns of behavioral intention and challenging the limitations of conventional linear frameworks. Methodologically, the integrated application of PLS-SEM, fsQCA, and NCA provides a rigorous and nuanced analytical framework for investigating sustainable consumption behaviors. Practically, the findings provide an evidence-based foundation upon which industry stakeholders can devise more effective marketing and strategic interventions to promote PBMA consumption.

## Figures and Tables

**Table 1:** Substantive and method factor loadings.

Indicators	Substantive Factor Loading (R_a_)	Substantive Variance ( $R_{a}^{2}$)	Method Factor Loading (R_b_)	Method Variance ( $R_{b}^{2}$)
EC1	0.752 ***	0.5655	−0.029	0.0008
EC2	0.862 ***	0.7430	0.050	0.0025
EC3	0.854 ***	0.7293	−0.002	0.0000
EC4	0.793 ***	0.6288	0.065	0.0042
HC1	0.813 ***	0.6610	−0.025	0.0006
HC2	0.843 ***	0.7106	0.074	0.0055
HC3	0.866 ***	0.7500	0.056	0.0031
HC4	0.843 ***	0.7106	0.100	0.0100
ATT1	0.907 ***	0.8226	0.038	0.0014
ATT2	0.921 ***	0.8482	−0.007	0.0000
ATT3	0.873 ***	0.7621	0.060	0.0036
SN1	0.894 ***	0.7992	0.021	0.0004
SN2	0.889 ***	0.7903	0.112	0.0125
SN3	0.898 ***	0.8064	0.022	0.0005
PBC1	0.890 ***	0.7921	0.065	0.0042
PBC2	0.894 ***	0.7992	0.079	0.0062
PBC3	0.856 ***	0.7327	0.054	0.0029
PI1	0.872 ***	0.7604	0.012	0.0001
PI2	0.924 ***	0.8538	0.023	0.0005
PI3	0.912 ***	0.8317	0.033	0.0011
Average	0.8678	0.7549	0.0401	0.0030

Note: *** *p* < 0.01.

**Table 2:** Results of measurement model.

Constructs	Items	VIF	Loadings	Cronbach’s α	rho_A	CR	AVE
Environmental Consciousness (EC)	EC1	1.503	0.762	0.833	0.837	0.889	0.667
	EC2	2.218	0.861				
	EC3	2.230	0.850				
	EC4	1.756	0.789				
Health Consciousness (HC)	HC1	1.823	0.814	0.863	0.865	0.907	0.708
	HC2	2.023	0.848				
	HC3	2.348	0.866				
	HC4	2.061	0.838				
Attitude (ATT)	ATT1	2.699	0.910	0.883	0.887	0.928	0.811
	ATT2	3.039	0.921				
	ATT3	2.144	0.870				
Subjective Norms (SN)	SN1	2.285	0.894	0.874	0.877	0.922	0.798
	SN2	2.377	0.887				
	SN3	2.387	0.899				
Perceived Behavior Control (PBC)	PBC1	2.240	0.891	0.854	0.858	0.911	0.774
	PBC2	2.227	0.893				
	PBC3	1.947	0.855				
Purchasing intention (PI)	PI1	2.072	0.877	0.887	0.888	0.930	0.815
	PI2	3.232	0.923				
	PI3	3.030	0.909				

Note: rho_A = consistent reliability coefficient; CR = composite reliability; AVE = average variance extracted.

**Table 3:** Results of discriminant validity.

	ATT	EC	HC	PBC	PI	SN
ATT	**0.901**	0.587	0.530	0.728	0.844	0.844
EC	0.505	**0.81** **6**	0.549	0.508	0.640	0.555
HC	0.465	0.471	**0.842**	0.449	0.512	0.490
PBC	0.631	0.429	0.389	**0.88** **0**	0.829	0.717
PI	0.748	0.554	0.448	0.725	**0.903**	0.870
SN	0.745	0.479	0.427	0.622	0.769	**0.894**

Note. ATT = Attitude, EC = Environmental consciousness, HC = Health consciousness, PBC = Perceived behavioral control, SN = Subject norms, PI = Purchase intention. The diagonal elements (in bold) represent the square roots of the AVE. The diagonal values were all greater than the corresponding off-diagonal elements in the lower half of the matrix, thereby confirming discriminant validity in accordance with the Fornell–Larcker criterion. Moreover, the heterotrait–monotrait (HTMT) ratios, shown in the off-diagonal elements of the upper triangular matrix, all fell below the conservative threshold of 0.9.

**Table 4:** Structural relationships.

					BCCI						
Hypothesis		Beta	t-Value	*p*-Value	5%	95%	Results	VIF	*f* ^2^	*R* ^2^	*Q* ^2^
Direct effect											
H1	EC → ATT	0.368	6.585	0.000	0.275	0.460	Supported	1.284	0.155	0.321	0.310
H2	EC → PI	0.141	3.850	0.000	0.080	0.201	Supported	1.528	0.049	0.735	0.691
H3	HC → ATT	0.292	5.270	0.000	0.201	0.382	Supported	1.284	0.098		
H4	HC → PI	0.014	0.033	0.739	−0.055	0.082	Not supported	1.428	0.001		
H5	ATT → PI	0.226	4.311	0.000	0.140	0.312	Supported	2.676	0.072		
H6	SN → PI	0.337	6.896	0.000	0.257	0.417	Supported	2.514	0.170		
H7	PBC → PI	0.306	7.233	0.000	0.237	0.377	Supported	1.855	0.191		
Indirect effect											
H8 EC → ATT → PI		0.083	3.541	0.000	0.050	0.129	Supported				
H9 HC → ATT → PI		0.066	3.405	0.000	0.039	0.104	Supported				

Note. BCCI = Bias-corrected confidence intervals. ATT = Attitude, EC = Environmental consciousness, HC = Health consciousness, PBC = Perceived behavioral control, SN = Subject norms, PI = Purchase intention.

**Table 5:** Calibration values for causal conditions and outcomes.

	Calibration Criteria		
Outcome/Conditions	95%	50%	5%
Environmental consciousness	5	4	3
Health consciousness	5	4.25	3
Attitude	5	4	3
Subject norms	5	3.667	3
Perceived behavioral control	5	4	2.667
Purchase intention	5	4	2.333

**Table 6:** Sufficient configurations of purchase intention towards PBMAs.

**Causal Models for High Purchase Intention Towards PBMAs**			
6A. PI = f(EC, HC, ATT, SN, PBC)	Raw Coverage	Unique Coverage	Consistency
Solution 1: ATT *~HC	0.488	0.009	0.800
Solution 2: ATT * PBC	0.794	0.022	0.881
Solution 3: ATT * SN	0.792	0.019	0.907
Solution 4: ~EC *~HC * SN	0.435	0.021	0.843
Solution 5: EC * HC *~PBC	0.377	0.007	0.846
Solution 6: EC * PBC *~SN	0.412	0.011	0.882
Solution Coverage	0.939		
Solution Consistency	0.772		
**Causal models for low purchase intention towards PBMAs**			
~6A. ~PI = f(EC, HC, ATT, SN, PBC)	Raw Coverage	Unique Coverage	Consistency
Solution 1: ~EC *~HC	0.672	0.061	0.880
Solution 2: EC *~SN	0.463	0.026	0.873
Solution 3: ~ATT *~PBC *~SN	0.648	0.046	0.965
Solution 4: EC * HC *~PBC	0.360	0.004	0.907
Solution 5: ATT *~EC * PBC	0.389	0.013	0.818
Solution 6: ATT *~PBC * SN	0.364	0.009	0.905
Solution Coverage	0.883		
Solution Consistency	0.818		

Note: The solutions were generated by applying a consistency threshold of 0.8 and a minimum frequency threshold of four cases per configuration; “~” indicates the absence or negation of a condition.; ATT = Attitude, EC = Environmental consciousness, HC = Health consciousness, PBC = Perceived behavioral control, SN = Subject norms, PI = Purchase intention. “*” indicates “and”.

**Table 7:** CE-FDH Derived Necessity Effect Sizes.

Constructs	Effect Size *d*	*p*-Value
Environmental consciousness	0.213	0.018
Health consciousness	0.101	0.049
Attitude	0.278	0.000
Subject norms	0.177	0.000
Perceived behavioral control	0.174	0.000

**Table 8:** Bottleneck table (percentage).

Purchase Intention	EC	HC	ATT	SN	PBC
0	NN	NN	NN	NN	NN
10	NN	NN	NN	NN	NN
20	NN	NN	NN	NN	NN
30	0.559	NN	0.559	NN	NN
40	0.559	NN	0.559	NN	NN
50	0.559	NN	0.559	NN	NN
60	0.559	1.117	3.073	NN	1.955
70	0.559	1.117	3.073	8.659	1.955
80	0.559	1.117	19.274	11.732	1.955
90	0.559	1.117	21.508	49.441	28.212
100	0.559	1.117	27.654	53.352	37.430

Note: ATT = Attitude, EC = Environmental consciousness, HC = Health consciousness, PBC = Perceived behavioral control, SN = Subject norms. NN = not necessary.

**Table 9:** Subsample’s intermediate configurational solutions (models).

	Raw Coverage	Unique Coverage	Consistency
Model 1: ATT * PBC *~SN	0.406	0.021	0.848
Model 2: ATT * EC *PBC	0.653	0.242	0.940
Model 3: ATT * EC *~HC *~SN	0.316	0.005	0.903
Model 4: ~ATT * EC *HC *~SN	0.325	0.048	0.839
Model 5: ATT *~EC *~HC * SN	0.387	0.039	0.897
Solution Coverage	0.809		
Solution Consistency	0.827		

Note: “~” denotes the absence or negation of a condition. “*” indicates “and”.

**Table 10:** Compare and contrast the results obtained from PLS-SEM, fsQCA, and NCA.

Constructs	PLS-SEM	fsQCA	NCA
Environmental consciousness	Environmental consciousness exerts a significant positive influence on both attitude and purchase intention.	Environmental consciousness (EC) appears in two of the six identified configurations (Solutions 5 and 6), suggesting that EC serves as a sufficient condition for high purchase intention towards PBMAs.	Environmental consciousness constitutes a necessary condition for achieving high purchase intention towards PBMAs and exerts a medium-level effect on purchase intention.
Health consciousness	Health consciousness demonstrates a positive effect on attitude; however, its direct influence on purchase intention is not statistically significant.	Health consciousness is present in one configuration (Solution 5) and is likewise identified as a sufficient condition for high purchase intention.	Health consciousness is identified as a necessary condition with a medium effect size.
Attitude	Attitude was found to be strongly and positively associated with purchase intention. Mediation analyses further reveal that attitude partially mediates the nexus between environmental consciousness and purchase intention, whereas it fully mediates the nexus between health consciousness and purchase intention.	Attitude emerges in three configurations (Solutions 1, 2, and 3), confirming its sufficiency in driving high purchase intention.	Attitude (ATT) demonstrates a medium effect on purchase intention. The analysis indicates that, to exhibit a strong purchase intention towards PBMAs, consumers must possess a minimum level of ATT equivalent to at least 20% of the threshold required for the formation of purchase intention.
Subject norms	Subjective norms are shown to exert significant positive effects on consumers’ purchase intention towards PBMAs.	Subjective norms appear in two configurations (Solutions 3 and 4), which are considered sufficient conditions for the emergence of high purchase intention.	Subjective norms (SN) display a medium effect on purchase intention, with consumers requiring a minimum threshold of approximately 12% of SN to attain strong purchase intentions.
Perceived behavioral control	Perceived behavioral control has a significant positive influence on consumers’ intention to purchase PBMAs.	Perceived behavioral control is present in two configurations (Solutions 2 and 6), which is considered sufficient conditions for the emergence of high purchase intention.	Perceived behavioral control is found to be a necessary condition with a medium effect on purchase intention.

## Data Availability

The datasets used and/or analyzed during the current study are available from the corresponding author on reasonable request at kunshan@mail.tku.edu.tw.

## Appendix A

**Table A1:** Study variables and items with means, standard deviations, skew and kurtosis.

Study Variables and Items	Mean	SD	Skew	Kurtosis
Environmental consciousness (EC) (Resource from [5])				
EC1. I am interested in environmental protection.	3.90	0.857	−0.610	0.788
EC2. I buy products that have a less harmful impact on the environment.	3.95	0.724	−0.547	0.981
EC3. I tend to be conscious of environmental issues when purchasing products.	3.79	0.804	−0.487	0.276
EC4. I buy products that have less impact on the environment if the products are otherwise the same.	4.08	0.728	−0.819	1.634
Health consciousness (HC) (Resource from [5])				
HC1. I choose food carefully for my health.	4.23	0.788	−0.815	0.340
HC2. I think about my health issues often.	4.15	0.704	−0.603	0.744
HC3. I pay a lot of attention to my health.	4.03	0.752	−0.523	0.132
HC4. I try to choose healthy foods.	4.11	0.732	−0.564	0.189
Attitude (AT) (Resource from [5])				
AT1. Choosing a plant-based meat alternative was a wise decision.	4.05	0.815	−0.436	−0.550
AT2. I am satisfied with choosing to eat plant-based meat alternatives.	4.00	0.818	−0.458	−0.216
AT3. Plant-based meat alternatives are trustworthy.	3.98	0.821	−0.513	0.082
Subjective norms (SN) (Resource from [26])				
SN1. My family and friends think I should choose to eat plant-based meat alternatives.	3.69	0.983	−0.460	−0.195
SN2. My superiors and colleagues support me when I buy plant-based meat alternatives.	3.51	0.907	−0.079	−0.070
SN3. Most people who matter to me believe I should purchase plant-based meat alternatives.	3.54	0.924	−0.086	−0.265
Perceived behavioral control (PBC) (Resource from [26])				
PBC1. I clearly know where to purchase plant-based meat alternatives.	3.94	0.859	−0.622	0.145
PBC2. I have knowledge of how to eat plant-based meat alternatives appropriately.	3.97	0.836	−0.660	0.483
PBC3. If I have the will, purchasing plant-based meat alternatives is easy.	4.00	0.820	−0.675	0.303
Purchase intention (PI) (Resource from [5])				
PI1. I plan to continue purchasing plant-based meat alternatives in the future.	3.94	0.845	−0.612	0.211
PI2. I would like to spread information about plant-based meat alternatives.	3.62	0.953	−0.211	−0.287
PI3. I would like to share product information about plant-based meat alternatives with my friends.	3.84	0.904	−0.663	0.406

Note: SD = standard deviation.
